# Sangyod rice bran extract enhances *Lacticaseibacillus paracasei* growth during the exponential phase and antibacterial activity of *L. paracasei* supernatant against zoonotic and foodborne pathogens

**DOI:** 10.14202/vetworld.2022.2466-2474

**Published:** 2022-10-28

**Authors:** Krittika Kabploy, Phirabhat Saengsawang, Chonticha Romyasamit, Suthinee Sangkanu, Warangkana Kitpipit, Thotsapol Thomrongsuwannakij, Tuempong Wongtawan, Mareena Daus, Maria de Lourdes Pereira, Watcharapong Mitsuwan

**Affiliations:** 1 School of Agricultural Technology and Food Industry, Walailak University, Nakhon Si Thammarat 80160, Thailand; 2 Center of Excellence in Innovation of Essential Oil and Bioactive Compounds, Walailak University, Nakhon Si Thammarat, 80160, Thailand; 3 Akkhraratchakumari Veterinary College, Walailak University, Nakhon Si Thammarat, 80160, Thailand; 4 One Health Research Center, Walailak University, Nakhon Si Thammarat, 80160, Thailand; 5 School of Allied Health Sciences and World Union for Herbal Drug Discovery (WUHeDD), Walailak University, Nakhon Si Thammarat, Thailand; 6 Division of Physical Science and Natural Products Center of Excellent, Faculty of Science, Prince of Songkla University, Songkhla, 80160, Thailand; 7 CICECO-Aveiro Institute of Materials and Department of Medical Sciences, University of Aveiro, Aveiro, 3810-193, Portugal; 8 Food Technology and Innovation Center of Excellence, Walailak University, Nakhon Si Thammarat, 80160, Thailand

**Keywords:** Avian pathogenic *Escherichia coli*, Bacillus cereus, Lacticaseibacillus paracasei, Sangyod rice bran extract, *Staphylococcus aureus*

## Abstract

**Background and Aim:**

Prebiotics are a group of nutrients or compounds that are degraded by the gut microbiota, including *Lacticaseibacillus paracasei*. The probiotic plays an important role in adhesion to the gut and is able to produce antimicrobial substances to inhibit pathogens. This study aimed to investigate the effects of Sangyod rice bran extract on the growth promotion of *L. paracasei*. Furthermore, antibacterial activity of the extract and *L. paracasei* supernatants cultured in De Man, Rogosa and Sharpe (MRS) medium plus the extract against zoonotic and foodborne pathogens was investigated.

**Materials and Methods:**

Antibacterial activity of the crude extract and the oil from Sangyod rice bran against the pathogens, including *Bacillus cereus*, *Staphylococcus aureus*, *Escherichia coli*, Avian pathogenic *E. coli*, and *Pseudomonas aeruginosa* was investigated using broth microdilution assay. The effects of the crude extract and the oil on the growth and adhesion of *L*. *paracasei* were further determined. The antibacterial activity of *L. paracasei* supernatant cultured in the medium supplemented with the extract and the oil against the pathogens was determined by agar well diffusion assay, followed by the broth microdilution assay. Finally, the chemical constituents and antioxidant activity of the crude extract and the oil from Sangyod rice bran were investigated.

**Results:**

The crude extract and the oil from Sangyod rice bran enhanced *L. paracasei* growth during the exponential phase. Furthermore, the crude extract at 0.25 mg/mL significantly enhanced the adhesion of *L. paracasei* to the surface compared with the control. Both minimal inhibitory concentration (MIC) and minimal bactericidal concentration (MBC) values of the crude extract against *B. cereus* and *S. aureus* were 0.5 and 1.0 mg/mL, respectively. All pathogens were sensitive to the supernatant of *L. paracasei* with similar MIC and MBC ranging from 12.5% v/v to 50% v/v. However, the MIC and MBC values of *L. paracasei* supernatant grown in MRS medium plus the crude extract and oil were not significantly different compared to the supernatant obtained from MRS alone. The crude extract had free radical scavenging activities with IC_50_ values at 0.61 mg/mL.

**Conclusion:**

The results suggested the potential benefits of the crude extract from Sangyod rice bran for inducing the growth and the adhesion of *L. paracasei* and inhibiting zoonotic and foodborne pathogens.

## Introduction

Zoonotic and foodborne pathogen-related infectious illnesses continue to be of great concern worldwide. *Escherichia coli* is a Gram-negative bacterium found as commensal microorganisms in the intestinal tract of humans and animals. However, some strains of *E. coli* are considered pathogens that cause fatal diseases in humans and animals [1]. Avian pathogenic *E. coli* (APEC) is a causative agent of colibacillosis in poultry, including chicken [2]. In addition, the infection caused by APEC is one of the leading causes of a decrease in meat (2% decline in live weight and 2.7% deterioration in feed conversion ratio) and egg production (up to 20%) [3, 4]. In addition, some Gram-positive bacteria, such as *Bacillus cereus* and *Staphylococcus aureus* have been well-known as foodborne and zoonotic pathogens. *Bacillus cereus* is a typical Gram-positive bacterium that results in foodborne bacterial intoxications. *Staphylococcus aureus* is one of the main causes of bovine mastitis in dairy cows and skin infection in animals. Notably, the pathogens have been suggested as a potential foodborne zoonotic pathogen that serves as an external source for human infections [5, 6]. Furthermore, the treatment of the pathogens is becoming difficult due to the increase in antibiotic resistance presented in the bacteria [7].

Probiotics are healthful microorganisms, including lactic acid bacteria, in many parts of the human and animal body, such as guts, oral cavity, and reproductive tract [8]. Lactic acid bacteria of the genus *Lactobacillus*, including *Lactobacillus paracasei*, are well-known as probiotics and belong to the normal mucosal microbiota of humans and animals [9]. Recently, *L. paracasei* has been renamed as *Lacticaseibacillus paracasei* due to the difference in the genome, compared to *Lactobacillus* [10]. Probiotics adhere to the surface of the gastrointestinal tract to protect the host from intestinal infection and maintain the stability of the gastrointestinal tract [11]. Recently, the antibacterial activity of the supernatant of *L. paracasei* against methicillin-resistant *S. aureus* (MRSA), *Listeria monocytogenes*, and *E. coli* O157:H7 was previously reported by our research team [12].

Prebiotics are a group of nutrients or compounds degraded by the gut microbiota, including probiotics. Beneficial intestinal probiotics utilize the substances, enhancing the probiotics’ growth and activity [13]. Sangyod Phattalung rice (*Oryza sativa* L.) is a red-violet pigmented rice originating in Phattalung Province, Southern part of Thailand. Sangyod rice is the first rice registered as a geographical indication product from Thailand. The substances in the rice, such as carbohydrates, proteins, vitamins B1, and B2, promote the growth of humans, animals, and bacteria [14]. The production of Sangyod rice generates some by-products, including rice bran is used as animal feed. An extract of Sangyod rice bran contains many bioactive compounds that have antioxidant and antidiabetic activities [14]. However, to the best of our knowledge, no studies on the effects of Sangyod rice bran extract as prebiotics to enhance the growth and activity of probiotics against pathogenic bacteria have been scientifically reported.

Therefore, this study aimed to investigate the effects of Sangyod rice bran extract on the growth promotion of *L. paracasei*. Furthermore, antibacterial activity of the extract and *L. paracasei* supernatants cultured in MRS medium plus the extract against zoonotic and foodborne pathogens was investigated. Finally, the antioxidant activity and chemical constituents of the extract were further investigated.

## Materials and Methods

### Ethical approval

This article does not contain any studies with human participants or animals performed by any authors.

### Study period and location

The present study was conducted from December 2021 to March 2022. Samples of bran of Sangyod rice were harvested and collected from a paddy field in Phatthalung Provinces, Southern Thailand. Extraction, antibacterial activity tests, and antioxidant determination of the extracts were carried out at the Laboratory of Bacteriology, Walailak University, Nakhon Si Thammarat, Thailand.

### Bacterial strains and bacterial growth condition

*Escherichia coli* ATCC 25922, *B. cereus* WU22001, *Pseudomonas aeruginosa* ATCC27853, and *S. aureus* ATCC25923 were used. All the pathogens were purchased from the American Type Culture Collection except APEC and *B. cereus* was kindly received from Associate Professor Veeranoot Nissapatorn, Walailak University. Five clinical isolates of APEC, including CH01, CH06, CH08, CH09, and CH10 were used. The bacteria were isolated from broilers and breeders with colibacillosis in Thailand, and identified as APEC as reported by our research team [7]. Five virulence-associated genes, including *iroN*, *ompT*, *hlyF*, *iss*, and *iutA* were detected using pentaplex PCR as previously described [7] to confirm as APEC. *Lacticaseibacillus paracasei* WU2502, a probiotic isolated from Palmyra palm sugar, was used as the probiotic strain as described by our research team [12]. All bacteria were cultured on Tryptic Soy Agar (TSA) (Difco, Claix, France) and incubated at 37°C for 24 h. Then, the microorganisms were grown in Tryptic Soy Broth (TSB) (Himedia, Nashik, India) at 37°C for 18–24 h, stored in TSB containing 25% glycerol at −80°C until use.

### Preparation of bran extract of Sangyod rice

Bran powder of Sangyod rice was obtained from a local rice mill in Phatthalung Province, Thailand. A rice milling machine was only used for Sangyod rice. Identification of Sangyod rice was carried out by Associate Professor Dr. Chatchai Kanlayanapaphon, Department of Biology, School of Sciences, Walailak University. The bran was extracted as previously described by our research team with minor modifications [15]. Briefly, a total of 300 g of the bran powder of Sangyod rice was solved in 500 mL of 95% ethanol for 7 days at 25–30°C. The respective liquid extracts were subsequently filtered through qualitative filter papers No 1, (Whatman International Ltd., Maidstone, UK). The solution was then concentrated under reduced pressure at 40°C using a rotary evaporator (Buchi Labortechnik AG, Flawil, Switzerland). The sample was air-dried at room temperature to eliminate the remainder of the solvent. The extract of the bran contained two parts, including oil on the top and crude extract at the bottom. The oil and the crude extract were separately prepared at 1000 mg/mL in 100% dimethyl sulfoxide (DMSO) and stored at 4°C until used.

### Effects of the crude extract and the oil on the growth of *L. paracasei*

The effects of the crude extract and the oil from Sangyod rice bran on the growth of *L. paracasei* were carried out as described [16]. An inoculum of *L. paracasei* (2 × 10^6^ Colony-forming unit [CFU]/mL) was cultured in TSB supplemented with the crude extract and/or the oil at a concentration of 0.25 mg/mL. The TSB medium supplemented with 1% DMSO was used as a negative control. Samples were incubated at 37°C and collected at 0, 6, 12, 18, and 24 h. At each time point, 100 mL of the sample was serially diluted in sterile phosphate-buffered saline (PBS). Viable bacteria were counted by a drop plate method in TSA, and incubated at 37°C for 24 h. The experiment was performed in triplicate. Data were presented as mean log numbers of viable cells ± standard deviation.

### Effects of the crude extract and the oil on the adhesion of *L. paracasei* to the 96-well microtiter plate

The effects of the crude extract and the oil on the adhesion of *L. paracasei* were investigated in a 96 well flat-bottom plate as described [15, 17] with modification. A total of 100 μL (2 × 10^6^ CFU/mL) of the bacterial cells cultured in an MRS medium was added to the wells of a sterile 96-well plate containing the extracts at different concentrations. In addition, the sterile broth containing 1%DMSO was included as the negative control. Samples were incubated at 37°C for 24 h. Then, the wells were washed with 200 μL of PBS buffer twice. Adherent bacteria were stained with 200 μL of 0.1% crystal violet for 30 min. The samples were rinsed with water and the wells were air-dried at room temperature. The dye bound to the adherent cells was dissolved with 200 μL 100% DMSO. To determine the inhibitory action of the crude extract and the oil, the biofilm formation was measured at an optical density (OD) of 570 nm by a Microtiter pate reader (Thermo-Scientific, Singapore City, Singapore). The results were presented as the mean of the OD 570 nm ± standard deviation.

### Antibacterial activity of the culture supernatant of *L. paracasei* against pathogens

The antibacterial activity of the culture supernatant of the probiotics against pathogens was determined by agar well diffusion assay as described [12, 16] with modification. Briefly, *L. paracasei* WU 2502 was cultured in MHB supplemented with the crude extract and/or the oil at 0.25 mg/mL, and incubated at 37°C for 24 h. Then, the samples were centrifuged at 5000 × g for 5 min to collect the supernatant. Samples were filtered through a sterile 0.45 μm filter (Pall Corporation, New York, USA) and kept at −20°C until use. The pathogens were cultured in MHB and incubated at 37°C for 4–6 h. The bacterial culture was adjusted to 0.5 McFarland standards. A total of 100 μL suspension were added and spread on MHA plates. Then, 8-mm-diameter wells were cut into the agar surface using the back of a sterile blue tip. One hundred microliters of the culture supernatant of the probiotics were loaded into each well. The culture medium and 3% hydrogen peroxide were included as negative and positive controls, respectively. In addition, the crude extract and the oil at 0.25 mg/mL were tested against the pathogens to confirm whether both extracts did not inhibit bacterial growth. The plates were incubated at 37°C for 24 h. Inhibition zones were measured using a Vernier caliper. The experiment was performed in triplicate. Data were presented as mean log numbers of viable cells ± standard deviation.

### Minimal inhibitory concentration (MIC) and minimal bactericidal concentration (MBC)

The MIC and MBC values of the crude extract and the oil from Sangyod rice bran and the supernatant of *L. paracasei* cultured in the medium supplemented with the crude extract and/or the oil against the pathogens were determined by broth microdilution assay as described by Scillato *et al*. [18] with modification. Briefly, 100 μL of the extracts and the filtered supernatant were added to a 96 well microtiter plate containing 100 μL of MHB and serially diluted two-fold using the MHB. The suspension of the tested bacteria (100 μL, 10^6^ CFU/mL) was added to each well and incubated at 37°C for 18 h. One percent DMSO was used as a negative control, while cefotaxime and vancomycin were used as the positive controls. Then, 0.03% of resazurin (Thermo Fisher Scientific, Lancashire, UK) was added and incubated for 3 h to determine MIC values. The MIC was defined as the lowest concentration that completely inhibited bacterial growth with a blue color. All wells shown in blue color were used to check MBC values by streaking the culture of the wells on TSA plates. The experiments were performed in triplicate.

### 1,1-Diphenyl-2-picrylhydrazyl (DPPH) free radical scavenging activity

The antioxidant activity of the crude extract and the oil from Sangyod rice bran was evaluated by DPPH free radical scavenging activity as described [19]. The 50% inhibition concentration (IC_50_) is defined as an efficient concentration of the sample necessary to reduce the initial DPPH concentration by 50%. The crude sample stock solution (200 mg/mL) was diluted to a final concentration of 0.2, 0.4, 0.6, 0.8, 1.0, and 1.2 mg/mL in ethanol. The oil sample stock solution (500 mg/mL) was diluted to final concentration of 40, 80, 120, 160, 200, and 240 mg/mL in ethanol. An aliquot of 80 μL of 0.1 mM DPPH ethanol solution was added to 20 μL of each sample solution and the final volume was adjusted to 500 μL with ethanol. The decrease in absorbance was measured at a wavelength of 515 nm over 0–20 min. Trolox was used as an external standard. Each sample was repeated 3 times. The percent inhibition was calculated following this equation;

Percent inhibition = 1-[(A _blank_-A _sample_)/A _blank_)] × 100

### Chemical constituents in the crude extract and the oil from Sangyod rice bran

To study the chemical components in the crude extract and the oil from Sangyod rice bran, gas chromatography-mass spectrometry (GC-MS) analysis was determined using an Agilent Technologies 7890 B (GC) equipped with a 5977A Mass Selective Detector (MS). Briefly, a VF-WAXms capillary column of dimensions 30 m × 250 × 0.25 μM was used with helium gas as a carrier at a flow rate of 1 mL/mL. The column temperature was initially programmed at 60°C, which was increased to 160°C at 10°C min^-1^. Then, the temperature was further increased to 250°C at 2.5°C min^-1^, held for 15 min. The mass spectrometer was performed in the electron ionization mode at 70 eV with a source temperature of 230°C, with continuous scanning from 35 to 500 m/z. The chemical constituents in the bran extracts, including the oil and the crude extract, were identified by comparing their mass spectral data with those from the Wiley library.

### Statistical analysis

All experiments were carried out in triplicate. The data were recorded, edited, and entered using the statistical package software (SPSS Inc. Chicago, IL, USA). The results were presented as mean ± standard deviation. Multiple comparisons between means were determined according to Duncan’s test. Furthermore, the two-tailed unpaired Student’s t-test was also analyzed. Differences were considered significant at p < 0.05.

## Results

### Extraction of Sangyod rice bran

**Figure-1:**
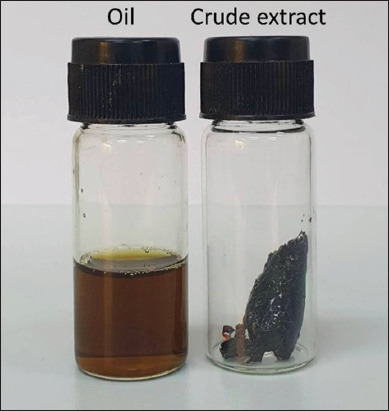
Physical appearance of crude extract and oil extracted from bran of Sangyod rice.

The dry powder of Sangyod rice bran was extracted with ethanol. The results showed that the extract of the bran contained two parts, including oil on the top and crude extract at the bottom (Figure-1). The percentage of yield values extracted from the crude extract and the oil was 0.47% and 2.60% per gram of dry weight of the bran, respectively.

### MIC and MBC values of the crude extract and the oil against the pathogens

The antibacterial activity of the crude extract and the oil from Sangyod rice bran against pathogens was determined by the broth microdilution assay. The crude extract showed antibacterial activity against the Gram-positive bacteria. Both MIC and MBC values of the crude extract against *B. cereus* and *S. aureus* were 0.5 and 1.0 mg/mL, respectively (Table-1). It was found that both the crude extract and the oil did not inhibit the growth of the tested Gram-negative bacteria, as observed at 4.0 mg/mL. The antibacterial activity of the positive antibiotics against the pathogens are presented in Table-1.

**Table-1:** MIC and MBC values of the crude extract and the oil from Sangyod rice bran against the pathogens.

Bacteria	MIC/MBC (mg/mL)			

	Crude extract	Oil	Ceftriaxone	Vancomycin
*B. cereus*	0.5/0.5	>4.0/>4.0	ND	0.001/0.002
*S. aureus*	1.0/1.0	>4.0/>4.0	ND	0.0005/0.001
*E. coli*	>4.0/>4.0	>4.0/>4.0	0.00025/0.00025	ND
APEC 09	>4.0/>4.0	>4.0/>4.0	0.00025/0.00025	ND
*P. aeruginosa*	>4.0/>4.0	>4.0/>4.0	0.001/0.002	ND

### The crude extract and the oil enhance the growth of *L. paracasei*

The prebiotic potential of the crude extract and oil obtained from Sangyod rice bran was determined in terms of their ability to stimulate the growth of a selected probiotic. The growth of *L. paracasei* in MRS medium, MRS plus the crude extract, and MRS plus the oil was determined. At 6 h, *L. paracasei* was significantly stimulated during the exponential phase by the addition of the crude extract and the oil, compared to the growth on MRS alone (Table-2). However, the growth of *L. paracasei* cultured under the three conditions was not significantly different during 12 and 24 h.

**Table-2:** Effects of the crude extract and oil from Sangyod rice bran on the growth of *L. paracasei.*

Time (h)	Mean ± SD (Log CFU/mL)		

	MRS + crude extract	MRS + oil	MRS alone
0	6.09 ± 0.02^a^	6.10 ± 0.02^a^	6.10 ± 0.02^a^
6	6.90 ± 0.01^b^	6.86 ± 0.02^b^	6.22 ± 0.02^a^
12	8.29 ± 0.08^a^	8.24 ± 0.09^a^	8.23 ± 0.05^a^
24	8.90 ± 0.01^a^	8.87 ± 0.02^a^	8.87 ± 0.02^a^

A significant difference was considered at p *<* 0.05

### Crude extract from Sangyod rice bran enhances the adhesion of *L. paracasei*

**Figure-2:**
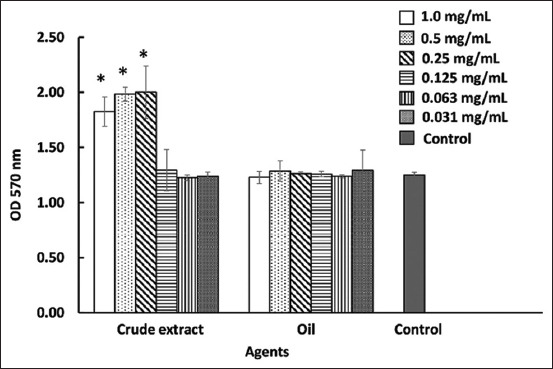
Effects of the crude extract and the oil from bran of Sangyod rice on the adhesion of *Lacticaseibacillus paracasei* WU2502. The bacteria were cultured in the medium supplemented with the crude extract and the oil at different concentrations, incubated at 37°C for 24 h. One percent DMSO was used as a negative control. The adhesion ability was observed at OD 570 nm. The data are presented as mean ± standard deviation (*significant difference; p < 0.05).

The activity of the crude extract and the oil on the adhesion of the probiotic was determined. The results demonstrated that the crude extract at 0.25 mg/mL significantly enhanced the adhesion of *L. paracasei* to the plastic surface compared with the control (p < 0.05) (Figure-2). However, the adhesion of the probiotic in the medium supplemented with the oil was not significantly different when compared with the control.

### Antibacterial activity of *L. paracasei* supernatant cultured in MRS medium plus the crude extract and the oil against pathogens

The antibacterial activity of *L. paracasei* supernatant grown in MRS medium supplemented with crude extract and oil was preliminarily tested by agar diffusion assay. Interestingly, all pathogens, including *E. coli*, APEC, *P. aeruginosa*, *B. cereus*, and *S. aureus* were sensitive to the supernatant of *L. paracasei* grown in MRS alone, MRS medium supplemented with the crude extract and oil (Table-3). The results demonstrated that the range of the inhibition zone of *L. paracasei* supernatant grown in the medium plus the crude extract and the oil against clinical isolates of APEC was 12.50 ± 0.50–16.67 ± 1.15 mm and 11.83 ± 0.76–16.00 ± 0.87 mm, respectively. Furthermore, the inhibition zones of the supernatant against other pathogens, including *E. coli*, *P. aeruginosa*, *B. cereus*, and *S. aureus*, were in the same range as the clinical isolates tested. However, the inhibition zone of *L. paracasei* supernatant grown in MRS medium plus the crude extract and oil was not significantly different compared to the supernatant obtained from MRS alone.

**Table-3:** Antibacterial activity of *L. paracasei* supernatant grown in MRS medium supplemented with the crude extract and oil against pathogens.

Pathogens	Inhibition zone (mm)			MIC/MBC (%v/v)		
	
	MRS + crude extract	MRS + oil	MRS alone	MRS + crude extract	MRS + oil	MRS alone
APEC CH01	12.50 ± 0.50	11.83 ± 0.76	11.83 ± 1.04	12.5/50	12.5/50	12.5/50
APEC CH06	14.00 ± 2.00	12.67 ± 0.58	13.33 ± 1.15	12.5/50	12.5/50	12.5/50
APEC CH08	13.00 ± 1.32	12.83 ± 0.29	12.50 ± 0.87	12.5/50	12.5/50	12.5/50
APEC CH09	16.67 ± 1.15	16.00 ± 0.87	15.33 ± 0.29	12.5/25	25/25	12.5/50
APEC CH10	13.67 ± 2.52	13.67 ± 1.53	12.33 ± 0.58	12.5/50	12.5/50	12.5/50
*E. coli*	12.67 ± 0.76	11.83 ± 0.29	12.50 ± 0.50	12.5/25	12.5/25	12.5/25
*P. aeruginosa*	16.33 ± 1.15	15.67 ± 1.53	14.67 ± 0.76	12.5/25	12.5/25	12.5/50
*B. cereus*	16.17 ± 1.44	15.33 ± 0.76	15.66 ± 1.15	12.5/12.5	12.5/25	12.5/50
*S. aureus*	12.33 ± 1.53	11.67 ± 1.15	11.17 ± 0.29	12.5/12.5	25/25	12.5/25

We further investigated the MIC and MBC values of the supernatant obtained from three different media against pathogens. The MIC and MBC values of *L. paracasei* supernatant cultured in MRS plus the crude extract, MRS plus the oil, and MRS alone against the pathogens were 12.5–25 and 25–50% v/v, respectively (Table-3).

### Antioxidant activity

**Figure-3:**
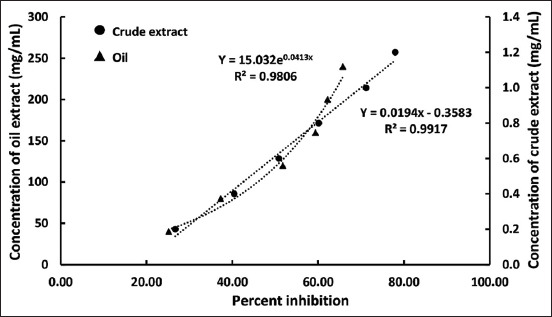
Antioxidant activity of the crude extract and the oil extracted from bran of Sangyod rice.

The antioxidant activity of the crude extract and oil was expressed as the DPPH concentration required a 50% absorbance reduction (IC_50_). The percentage inhibition curve for the DPPH radical-scavenging activity of the crude extract and oil and the IC_50_ values are presented in Figure-3. The crude extract had a free radical scavenging activity by decreasing the concentration of DPPH radicals. The IC_50_ value of the crude extract was 0.61 mg/mL. Meanwhile, the oil extract had a slightly potential scavenging activity on DPPH with the IC_50_ value of oil extract of 118.50 mg/mL.

### Chemical constituents in the crude extract and oil

**Figure-4:**
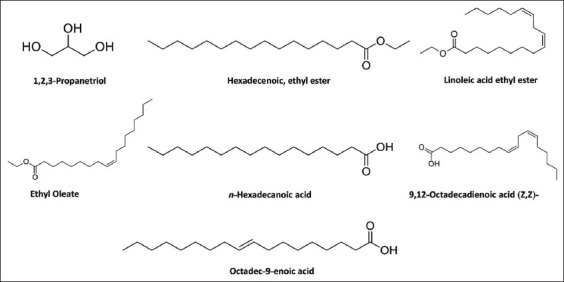
Chemical structures of pure compounds presented in the crude extract and oil from Sangyod bran as detected by gas chromatography-mass spectrometry analysis.

The identification of the chemical constituents in the crude extract and the oil was determined by GC-MS analysis. A total of 16 (Table-4) and 32 (Table-5) compounds were presented in the crude extracts and oil, respectively. As shown in Table-4 and Figure-4, 1,2,3-propanetriol was found as the main compound in the crude extracts, followed by hexadecanoic acid, ethyl ester, ethyl oleate, and linoleic acid ethyl ester, respectively. In contrast, 9,12-octadecadienoic acid was the main compound in the oil, followed by hexadecanoic acid, ethyl ester, ethyl oleate, 9-octadecenoic acid (Z)-, 2,3-dihydroxypropyl ester, and octadec-9-enoic acid, respectively.

**Table-4:** Chemical composition of the crude extract of Sangyod rice bran.

Component RT	Percentage of total	Formula	Compound name
9.5046	30.10	C_3_H_8_O_3_	1,2,3-Propanetriol
11.0547	0.13	C_6_H_8_O_4_	4H-Pyran-4-one, 2,3-dihydro-3,5-dihydroxy-6-methyl-
15.5179	5.19	C_10_H_13_N_5_O_5_	Guanosine
17.7268	2.20	C_8_H_16_O_6_	Ethyl. alpha-d-glucopyranoside
19.4317	0.70	C_16_H_32_O_2_	Tetradecanoic acid, ethyl ester
19.5913	1.92	C_9_H_8_O_3_	2-Hydroxy-5-methylisophthalaldehyde
21.2810	3.19	C_16_H_32_O_2_	n-Hexadecanoic acid
21.7243	11.60	C_18_H_36_O_2_	Hexadecanoic acid, ethyl ester
23.8116	2.06	C_18_H_32_O_2_	9,12-Octadecadienoic acid (Z, Z)-
23.9104	6.31	C_18_H_34_O_2_	Octadec-9-enoic acid
24.2778	8.16	C_20_H_36_O_2_	Linoleic acid ethyl ester
24.3715	10.79	C_20_H_38_O_2_	Ethyl Oleate
24.7820	0.91	C_20_H_40_O_2_	Octadecanoic acid, ethyl ester
30.1475	0.13	C_19_H_38_O_4_	Hexadecanoic acid, 2-hydroxy-1-(hydroxymethyl) ethyl ester
33.2457	0.41	C_21_H_38_O_4_	(Z, Z)-9,12-octadeca-dienoic acid, 2,3-dihydroxypropyl ester
33.3268	1.72	C_21_H_40_O_4_	9-Octadecenoic acid (Z)-, 2,3-dihydroxypropyl ester

**Table-5:** Chemical composition of the oil of Sangyod rice bran.

Component RT	Percentage of total	Formula	Compound name
11.9138	0.01	C_10_H_20_O_2_	Octanoic acid, ethyl ester
11.9619	0.01	C_12_H_26_	Dodecane
12.8991	0.01	C_10_H_18_O	2-Decenal, (E)-
14.8066	0.01	C_14_H_30_	Tetradecane
19.0599	0.02	C_14_H_28_O_2_	Tetradecanoic acid
19.4298	0.21	C_16_H_32_O_2_	Tetradecanoic acid, ethyl este
19.9796	0.01	C_18_H_36_O	2-Pentadecanone, 6,10,14-trimethyl-
20.4863	0.02	C_17_H_34_O_2_	Pentadecanoic acid, ethyl ester
20.8562	0.01	C_17_H_34_O_2_	Hexadecanoic acid, methyl ester
21.4870	9.58	C_16_H_32_O_2_	n-Hexadecanoic acid
21.7962	13.87	C_18_H_36_O_2_	Hexadecanoic acid, ethyl ester
23.1898	0.06	C_19_H_38_O_2_	Heptadecanoic acid, ethyl ester
23.3089	0.01	C_19_H_36_O_2_	8-Octadecenoic acid, methyl ester, (E)-
24.1856	19.41	C_18_H_32_O_2_	9,12-Octadecadienoic acid (Z, Z)-
24.2870	11.78	C_18_H_34_O_2_	Octadec-9-enoic acid
24.3732	9.79	C_20_H_36_O_2_	Linoleic acid ethyl ester
24.4923	12.85	C_20_H_38_O_2_	Ethyl Oleate
24.8293	1.62	C_20_H_40_O_2_	Octadecanoic acid, ethyl ester
27.7714	0.33	C_23_H_44_O_2_	n-Propyl 11-eicosenoate
28.2275		C_22_H_44_O_2_	Eicosanoic acid, ethyl ester
30.1838	0.65	C_19_H_38_O_4_	Hexadecanoic acid, 2-hydroxy-1-(hydroxymethyl) ethyl ester
31.7423	0.19	C_24_H_48_O_2_	Docosanoic acid, ethyl ester
33.4403	12.67	C_21_H_40_O_4_	9-Octadecenoic acid (Z)-, 2,3-dihydroxypropyl ester
33.7849		C_21_H_42_O_4_	Octadecanoic acid, 2,3-dihydroxypropyl ester
35.1915	0.14	C_26_H_52_O_2_	Ethyl tetracosanoate
39.8903	0.08	C_31_H_52_O_2_	.beta.-Sitosterol acetate
41.1424	0.18	C_30_H_50_O_2_	Betulin
41.8902	0.30	C_28_H_48_O	(3methyl, 24R)-ergost-5-en-3-ol
42.3997	0.55	C_29_H_48_O	Stigmasta-5,22-dien-3-ol, (3.beta.,22E)-
43.2716	1.71	C_29_H_50_O	24(Z)-methyl-25-homocholesterol
43.5251	0.06	C_29_H_48_O	Stigmasta-5,24 (28)-dien-3-ol, (3.beta.,24Z)-
45.2107	0.81	C_31_H_52_O	9,19-Cyclolanostan-3-ol, 24-methylene-, (3.beta.)-

## Discussion

Prebiotics are a group of nutrients or compounds that are degraded by the gut microbiota, including probiotics. The beneficial intestinal probiotics utilize the substances, resulting in enhanced growth and activity of the probiotics [13]. In this study, we showed the potential activity of the crude extract and oil from Sangyod rice bran to enhance the growth of *L. paracasei*, the probiotic candidate. Furthermore, the supernatant of *L. paracasei* cultured in the medium plus the crude extract and oil against zoonotic and foodborne pathogens were determined.

Adding the crude extract and the oil from Sangyod rice bran to the medium enhanced the growth of *L. paracasei* in 6 h of incubation compared to the control. It was well-accepted that this incubation time is the early exponential phase of the growth of *Lactobacillus* spp. such as *L. rhamnosus* [20]. The analysis of GC-MS of the crude extract and the oil from Sangyod rice bran demonstrated the presence of 48 total compounds with different chemical groups, such as fatty acids, esters, alcohols, carboxylic acids, and hydrocarbons. It was observed that members of ester groups, including fatty acids were found to be predominant in the extract and the oil. According to the results, 1,2,3-propanetriol, also known as glycerol, was the main compound in Sangyod rice bran crude extract. It has been reported that *Clostridium butyricum*, *Lactococcus garvieae*, *Lactobacillus brevis* [21], and *Klebsiella pneumoniae* [22] may use glycerol as a sole carbon source for the growth and potential production of 1,3-propanediol. This may be one of the possible mechanisms of actions of the extract in enhancing the growth of the probiotic.

Interestingly, the crude extract also enhanced the adhesion of the probiotic to the plastic surface. This may result from the enhanced growth of the probiotic by the crude extracts during the initial exponential phase. It has been reported that *L. paracasei* displayed the capacity to efficiently adhere to cells such as Caco-2 colon cancer cells as observed at 4 h [23]. Similarly, resveratrol enhanced the adhesion of *L. paracasei* to plastic surfaces and intestinal epithelial cells within 1.5 h [24]. Furthermore, resveratrol promoted biofilm formation by modifying the physicochemical properties of the surface of *L. paracasei*, hence promoting its adhesion capacity [24]. It is well-known that the adhesion of the probiotic to gastrointestinal tract can prevent the colonization of pathogens. It is important to emphasize that Sangyod rice bran crude extracts have antioxidant activity due to the high content of 1,2,3-propanetriol with hydroxyl groups. It has also been reported that a fraction of 1,2,3-propanetriol demonstrated high free radical scavenging properties compared to biodiesel and oil material [25].

The results revealed that the crude extract has bactericidal activity against *B. cereus* and *S. aureus* due to the MBC/MIC ratio [26]. Hexadecanoic acid has been reported to exhibit bactericidal activity against *S. aureus*, acting through disruption of the proton motive force, an increase in membrane fluidity, and its effects on electron transfer [27]. In addition, high contents of fatty acids and esters such as hexanoic acid and (Z, Z)-9,12-octadecadienoic acid present in *Helleborus bocconei* extract were considered as antimicrobial agents against pathogens including *B. subtilis*, *S. aureus*, *E. coli*, and *P. aeruginosa* [28]. However, the highest concentration tested of the crude extract and the oil from Sangyod rice bran did not inhibit the growth of Gram-negative pathogens. It has been accepted that the bacterial cell envelope of Gram-negative bacteria is different when compared to Gram-positive bacteria. Gram-negative bacteria contain an outer membrane; the latter lacks a low permeability barrier [29]. In addition, some hydrophobic compounds such as phenols, esters, and tannins are challenging to absorb into the outer membrane of Gram-negative pathogens, which are composed of phospholipids [30].

To overcome the limitation of the antibacterial activity of the crude extract from Sangyod rice bran, we used the crude extract as a growth-inducing agent to stimulate *L. paracasei*. Subsequently, *L. paracasei* supernatant cultured in the medium plus the extract was tested against zoonotic and foodborne pathogens. The results revealed that the pathogens were sensitive to the probiotic supernatant. However, it was observed that the crude extract and the oil did not potentiate the antibacterial activity of the supernatant. The antibacterial activity obtained from the results remained the bioactive compounds in the probiotic supernatant. In addition, synergistic effects between the probiotic supernatant and the crude extract were not observed. Recently, the antibacterial activity of the supernatant of *L. paracasei* WU2502 against pathogens, including *E. coli* and *E. coli* O157:H7 has been reported [12]. Furthermore, the probiotic supernatant exhibited anti-biofilm activity against *S. aureus* and MRSA [12].

The results indicated that the crude extract from Sanyod rice bran may be used as a prebiotic to enhance the growth and adhesion of the probiotic. However, it did not increase the antibacterial activity of the supernatant. The limitations of this study are the isolation of pure compounds from *L. paracasei* supernatant, which must be investigated. Subsequently, the antibacterial modes of action of *L. paracasei* supernatant and its pure compounds against the pathogens should be monitored. Further study should be performed *in vivo*/*ex vivo* to provide insight into the mechanism of the *L. paracasei* supernatant and pure compounds against the pathogens. Notably, the use of Sangyod rice bran crude extract as the prebiotic in animal feeds should be supported on a larger scale.

## Conclusion

The present study presented that the crude extract and the oil from Sangyod rice bran enhanced *L. paracasei* growth during the exponential phase. Furthermore, the crude extract at 0.25 mg/mL significantly enhanced the adhesion of *L. paracasei* to the surface compared with the control. Both MIC and MBC values of the crude extract against *B. cereus* and *S. aureus* were 0.5 and 1.0 mg/mL, respectively. Interestingly, all pathogens were sensitive to the supernatant of *L. paracasei*. However, the MIC and MBC values of *L. paracasei* supernatant grown in MRS medium plus the crude extract and oil were not significantly different compared to the supernatant obtained from MRS alone. The crude extract had free radical scavenging activities with IC_50_ values at 0.61 mg/mL. The results suggested the potential benefits of the crude extract from Sangyod rice bran for inducing the growth and the adhesion of *L. paracasei* and inhibiting the pathogens.

## Authors’ Contributions

KK, PS, WK, and WM: Conceived and designed the experiments. KK, PS, CR, SS, MD, and WM: Performed the experiments. KK, PS, WK, and WM: Analyzed and interpreted the data. KK, WK, PS, and WM: Statistical analysis. TW, TT, and MLP: Contributed reagents, materials, analysis tools, and data. KK, PS, MLP, and WM: Wrote the paper. All authors have read and approved the final manuscript.
